# Navigated Pattern Laser System versus Single-Spot Laser System for Postoperative 360-Degree Laser Retinopexy

**DOI:** 10.1155/2016/9871976

**Published:** 2016-12-14

**Authors:** Alexei N. Kulikov, Dmitrii S. Maltsev, Ernest V. Boiko

**Affiliations:** ^1^Department of Ophthalmology, Military Medical Academy, 5 Klinicheskaya St., St. Petersburg 194044, Russia; ^2^St. Petersburg Branch of the Academician S. Fyodorov IRTC “Eye Microsurgery”, 21 Yaroslav Gashek St., St. Petersburg 192283, Russia; ^3^Department of Ophthalmology, Mechnikov North-West State Medical University, 47 Kirochnaya St., St. Petersburg 191015, Russia

## Abstract

*Purpose*. To compare three 360°-laser retinopexy (LRP) approaches (using navigated pattern laser system, single-spot slit-lamp (SL) laser delivery, and single-spot indirect ophthalmoscope (IO) laser delivery) in regard to procedure duration, procedural pain score, technical difficulties, and the ability to achieve surgical goals.* Material and Methods*. Eighty-six rhegmatogenous retinal detachment patients (86 eyes) were included in this prospective randomized study. The mean procedural time, procedural pain score (using 4-point Verbal Rating Scale), number of laser burns, and achievement of the surgical goals were compared between three groups (pattern LRP (Navilas® laser system), 36 patients; SL-LRP, 28 patients; and IO-LRP, 22 patients).* Results*. In the pattern LRP group, the amount of time needed for LRP and pain level were statistically significantly lower, whereas the number of applied laser burns was higher compared to those in the SL-LRP group and in the IO-LRP group. In the pattern LRP, SL-LRP, and IO-LRP groups, surgical goals were fully achieved in 28 (77.8%), 17 (60.7%), and 13 patients (59.1%), respectively (*p* > 0.05).* Conclusion*. The navigated pattern approach allows improving the treatment time and pain in postoperative 360° LRP. Moreover, 360° pattern LRP is at least as effective in achieving the surgical goal as the conventional (slit-lamp or indirect ophthalmoscope) approaches with a single-spot laser.

## 1. Introduction

Three hundred sixty-degree laser retinopexy (360°-LRP) is an essential element in the treatment of complicated retinal detachment. The incidence of retinal redetachment has been shown to be reduced more than twofold (from as high as 26% to 14%) [1, 2] with prophylactic intraoperative 360°-LRP after removal of silicone oil. In addition, the rate of postvitrectomy RRD due to iatrogenic breaks has been significantly reduced (from 5.7% to 0%) with 360°-LRP [3]. Koh and colleagues have reported that intraoperative 360°-LRP following vitrectomy shows an encouraging reduction (approximately 74%) in the rate of postoperative retinal detachment [4]. A number of studies have demonstrated improved outcomes with the use of 360°-LRP in combined surgery for a giant retinal tear [5–7], with a reduction from 26% to 7% [7] in the rate of postoperative retinal detachment.

The 360° laser retinopexy is most commonly performed at the end of the vitrectomy with the endolaser probe [2–6, 8]. The drawbacks of intraoperative 360°-LRP are increased total operating time and the need for scleral depression to coagulate superior retinal locations which are hard to access. The use of intraoperative 360°-LRP technique may be additionally limited by cases of vitrectomy-related failures and complications, when it is not possible to achieve a complete retinal reattachment intraoperatively. Therefore, when used before the surgical procedure [9] or postponed completely for some time after this procedure, 360°-LRP may provide some advantages over intraoperative 360°-LRP. Moreover, 360°-LRP sometimes cannot be performed (in required cases) during the scleral buckling due to failure to achieve complete retinal reattachment caused by residual subretinal fluid.

Various modifications of conventional postoperative 360°-LRP with a single-spot laser attached to indirect ophthalmoscope or slit-lamp are laborious (since they involve the application of numerous laser burns around the entire fundus periphery) and painful due to postoperative ocular irritation.

Currently, the need for massive laser photocoagulation sessions is satisfied by using the pattern laser photocoagulation techniques. The laser parameter range available with the navigated pattern technology contributes to decreased pain and duration of laser photocoagulation procedure [10, 11]. Therefore, the pattern approach may make postoperative 360°-LRP less laborious and better tolerated by the patient.

The study purpose was to compare three 360°-LRP approaches (using navigated pattern laser system, single-spot slit-lamp laser delivery, and single-spot indirect ophthalmoscope laser delivery) in regard to (1) procedure duration, (2) procedural pain score, and (3) technical difficulties and the ability to achieve surgical goals.

## 2. Materials and Methods

The study was approved by the Ethics Committee of the Military Medical Academy and followed the tenets of the Declaration of Helsinki. All patients gave written informed consent both for participation in the study and for LRP.

Rhegmatogenous retinal detachment patients with clinical indications for laser retinopexy were included in this single-center prospective randomized longitudinal interventional study. LRP was indicated to prevent retinal redetachment after the surgical procedures specified in Table 1. In vitrectomy cases, 360°-LRP was performed to reduce the risk of retinal redetachment due to iatrogenic breaks [3, 4, 8]. In silicone tamponade cases, 360°-LRP was performed to reduce the risk of retinal redetachment after removal of silicone oil [2]. Some of these vitrectomy cases and silicone tamponade cases also underwent circular scleral buckling (CSB). In patients who underwent CSB only, 360°-LRP was performed due to a giant (>90°) tear, multiple retinal tears (with a total extension of 90° or more), or retinal dialysis. Previously, LRP has been reported to be effective in CSB for a giant retinal tear [5–7]. Since retinal dialysis and multiple retinal tears (with a total extension of 90° or more) are the two pathologies similar to the above, we used LRP also in relevant cases. All these cases correspond to the patients included in the study for a “circular scleral buckling-” relevant indication (Table 1).

Exclusion criteria were (1) incomplete performance of intraoperative LRP (excluding the cases when endolaser photocoagulation was applied outside the 360°-LRP site); (2) acute infections of the posterior segment; (3) postoperative inflammatory response; or (4) use of nonsteroidal anti-inflammatory, antihistamine, sedative, or other drugs which can potentially influence pain self-assessment.

### 2.1. Surgical Technique

Pattern laser retinopexy was performed using Navilas 532 laser system (OD-OS, Berlin, Germany) incorporating navigated Rapid PRP technology to produce 30 ms pulses, with square pattern from 3 × 3 to 5 × 5 laser spots (spot size, 450 *μ*m; spot spacing, 1 spot size). The Navilas wide-field Rapid PRP (Ocular Instruments, Inc., Bellevue, WA, USA) contact lens was used to deliver laser energy to the posterior segment.

Slit-lamp LRP (SL-LRP) was performed with the 532 nm GYC-1000 laser (NIDEK, Japan) attached to ophthalmic YAG laser system YC-1800 (NIDEK). A wide-field contact lens, Mainster PRP 165 (Ocular Instruments, Inc., Bellevue, WA) and/or G-3 Three-Mirror Glass Gonio Fundus Lens (Volk Optical, Inc., Mentor, OH) were used for laser delivery. Given the laser spot magnification of the lens, the actual retinal laser spot size was 350 *μ*m. A 1.0 burn-width spot spacing was used for all SL-LRP cases.

Indirect ophthalmoscope LRP (IO-LRP) was performed with the binocular indirect ophthalmoscope NBO-3-01 (ZOMZ, Sergiev Posad, Russia) and 532 nm GYC-1000 laser (NIDEK). A 20-dioptre noncontact aspheric lens (Ocular Instruments) was used for laser delivery. Given the laser spot magnification of the lens, the actual retinal laser spot size was 800 to 1000 *μ*m. A 1.0 burn-width spot spacing was used for all SL-LRP cases.

Patients were randomly assigned to the pattern LRP, SL-LRP, or IO-LRP.

### 2.2. Primary and Secondary Endpoints

Primary endpoints were amount of time needed for LRP, number of sessions, pain level, number of applied laser burns, and rate of surgical goal achievement. Retinal redetachment rate after silicone oil removal or after vitrectomy and/or buckling surgery was a secondary endpoint.

The procedural time was measured as the time that elapsed between the initial placement of the contact lens onto the eye (visualization of the fundus with the help of the 20-D aspheric lens in the IO-LRP group) and the final laser burn application, irrespective of the number of placements of the contact lens.

A session was defined as the LRP procedure performed during a patient's visit to the clinic. At the end of each session, the ophthalmologist made a decision whether the next session was required. If the next session was required, the date was scheduled based on the cause of a failure to complete a 360°-LRP within the first session (cases requiring resorption of subretinal fluid or improvement in vitreous clarity were given increased session-to-session intervals compared to those with difficulties associated with apparent pain, narrow pupil, fibrosis of the capsular bag, or decentration of the intraocular lens).

The total number of laser spots delivered in each patient during all LRP sessions was determined after completion of each session.

The 4-point Verbal Rating Scale (VRS; 0, no pain; 1, mild pain; 2, moderate pain; and 3, severe pain) was used to self-assess the procedural pain immediately following the procedure, with the patient being explained that the pain sensation was caused not by mechanical effects of the lens, but by exposure to laser irradiation [12].

**Figure 1:**
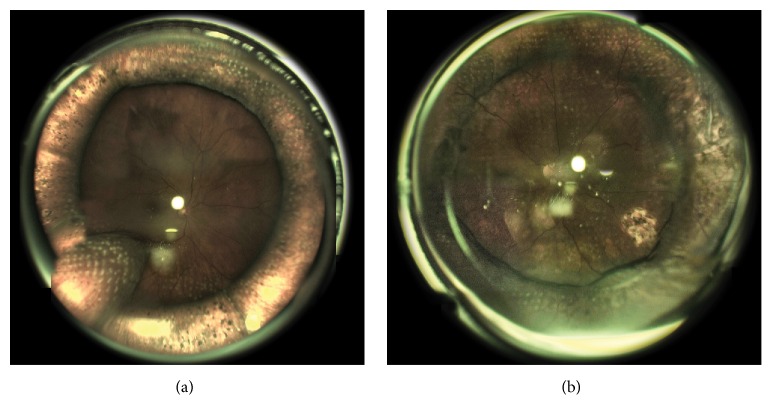
(a) Fundus image of the patient with a circular scleral buckle (CSB) and a meridional scleral buckle for multiple retinal tears, after two LRP sessions, with the surgical goal fully achieved. (b) Fundus image of the patient with a CSB and silicone oil tamponade of the vitreous cavity, after a single “pattern LRP” session, with the surgical goal fully achieved (LRP at a single tear was performed intraoperatively).

In each case, irrespective of the presence or absence of a tamponade of the vitreous cavity, the single surgical goal was to achieve either coagulation of the extreme and midperipheral fundus (with retinal tear photocoagulation) over 360 degrees or coagulation spread over the entire posterior slope of the buckle and anteriorly of it (with retinal tear photocoagulation) (Figure 1). In retinal tear photocoagulation a 0.5 burn-width spot spacing was used. The goal was considered not achieved if laser spots were not placed at a part of the retinal site planned for treatment with 360°-LRP.

Medical records were retrospectively reviewed to determine the times between the retinal detachment surgery and LRP separately for cases with and without silicone tamponade.

The technical difficulties encountered during each of the LRP session were assessed to explain the causes of possible difference in success rate of surgical goals among the study groups. Technical difficulty was defined as the presence of any condition (e.g., subretinal fluid and IOL decentration) hampering the placement and visual assessment of laser burns at a fundus site during its sequential photocoagulation in a clockwise fashion.

### 2.3. Follow-Up

Patients underwent fundus examinations at week 2 (following either silicone oil removal from the vitreous cavity or LRP after vitrectomy and/or buckling surgery) and then every month thereafter to exclude retinal redetachment after LRP. Minimum follow-up time was 6 weeks (with 2 consecutive visits). The presence of subretinal fluid posteriorly of the laser coagulation area was considered as retinal redetachment and was checked visually; OCT was done in doubtful cases.

### 2.4. Statistics

All data are presented as mean ± standard deviation. A one-way analysis of variance (ANOVA) with Bonferroni-adjusted post hoc comparisons was used to assess between-group differences in age and qualitative and quantitative characteristics of LRP. A chi-square test was used to assess between-group differences in male-to-female ratio, rate of surgical goal achievement, technical difficulty rate, and retinal redetachment rate.

## 3. Results

### 3.1. Demographics and Basic Characteristics of Subgroups

Eighty-six individuals (44 men and 41 women) were included in the study. There was no statistically significant difference in age and male-to-female ratio among the groups (Table 2).

### 3.2. Primary Endpoints Analysis

In the pattern LRP group, the amount of time needed for LRP, number of sessions, and pain level were statistically significantly lower, whereas the number of applied laser burns was higher compared to those in the SL-LRP group and in the IO-LRP group (*p* < 0.05) (Table 3). No statistically significant difference was found in these variables between the SL-LRP group and the IO-LRP group. In the pattern LRP group, SL-LRP group, and IO-LRP group, surgical goals were fully achieved in 28 patients (77.8%), 17 patients (60.7%), and 13 patients (59.1%), respectively, and not achieved (due to technical difficulties) in 12 patients, 11 patients, and 9 patients, respectively. There was no statistically significant difference in the rate of surgical goal achievement among the groups.

There was no statistically significant difference in mean time after retinal detachment surgery (in case of silicone oil tamponade, after silicone oil injection) among the groups (Table 3).

### 3.3. Technical Difficulties

In the pattern LRP group, technical difficulties were encountered in 18 cases (50.0%), with the most common difficulty being residual retinal detachment (11 cases), followed by the rigid pupil (which hampered visualization of the peripheral fundus; 5 cases), fibrosis of the capsular bag and/or decentered IOL (5 cases), irregularly placed CSB (anteriorly displaced; 2 cases), and media opacification (partial vitreous hemorrhage) early following retinal detachment surgery (1 case). The pattern LRP was aborted due to high pain levels in one patient.

In the SL-LRP group, technical difficulties occurred in 15 cases (53.6%) and included residual retinal detachment (6 cases), narrow and/or decentered pupil (5 cases), fibrosis of the capsular bag (2 cases), and posttraumatic corneal scar (1 case). In addition, the SL-LRP was aborted due to high pain levels and significant procedural time needed to fully achieve the surgical goal in 9 patients.

Technical difficulties were encountered in 12 cases (54.6%) of the IO-LRP group and included residual retinal detachment (5 cases), narrow pupil (4 cases), and fibrosis of the capsular bag and/or decentered IOL (3 cases). In addition, the IO-LRP was aborted due to high pain levels and significant procedural time needed to fully achieve the surgical goal in 5 patients.

There was no statistically significant difference in technical difficulty rate among the groups.

If a residual detachment was present at the extreme peripheral fundus, LRP was performed posterior of the detachment. These cases corresponded to “failure to achieve surgical goal.” Performing photocoagulation posterior of the area initially planned for photocoagulation was not included into the definition of the achievement of surgical goal, since it does not correspond to the definition of classical LRP.

### 3.4. Reasons for Failures to Achieve Surgical Goals

In the pattern LRP group, failures to achieve surgical goals were associated with residual retinal detachment (9 patients) or narrow pupil with fibrosis of the capsular bag and decentration of the intraocular lens (3 patients). In the SL-LRP group, these failures were associated with residual retinal detachment (6 patients), narrow pupil and fibrosis of the capsular bag (4 patients), or corneal scar (1 patient). In the IO-LRP group, failures to achieve surgical goals were associated with residual retinal detachment (5 patients) or narrow pupil with fibrosis of the capsular bag and decentration of the intraocular lens (4 patients).

### 3.5. Follow-Up after Silicone Oil Removal

In the pattern LRP, SL-LRP, and IO-LRP groups, the mean duration of follow-up after silicone oil removal was 6.6 ± 3.1 months, 8.1 ± 4.5 months, and 7.1 ± 4.1 months, respectively (ANOVA3x, *p* = 0.35), with redetachment found in 1 case (8.3%), 2 cases (18.2%), and 1 case (11.1%), respectively. No statistically significant difference was found in retinal redetachment rate after silicone oil removal among the groups (chi-square test, *p* = 0.77).

### 3.6. Follow-Up after Vitrectomy and/or Buckling Surgery

In the pattern LRP, SL-LRP, and IO-LRP groups, the mean duration of follow-up after vitrectomy and/or buckling surgery was 6.6 ± 3.4 months, 5.9 ± 4.0 months, and 6.3 ± 3.4 months, respectively (ANOVA3x, *p* = 0.44), with redetachment found in 1 case (4.2%), 1 case (5.9%), and no cases, respectively. No statistically significant difference was found in retinal redetachment rate after vitrectomy and/or buckling surgery among the groups (chi-square test, *p* = 0.70).

## 4. Discussion

The present study shows that 360°-LRP performed using the navigated pattern laser (Navilas) is less time-consuming and less painful than that performed with a single-spot laser coupled with a slit-lamp or indirect ophthalmoscope laser delivery system. In addition, there was no statistically significant difference in rate of full achievement of the surgical goal and in retinal redetachment rate between the pattern LRP and conventional 360° LRP techniques. The absence of significant difference in goal achievement rate can be explained by similar number and configuration of the technical difficulties (e.g., rigid pupil and residual detachment) resulting from the anatomic status of the eye after retinal detachment surgery and hampering photocoagulation irrespective of the method of laser delivery. However, the rate of achievement of the surgical goal in the pattern LRP was higher than in comparison groups, although not statistically significantly.

The effect of 360° LRP (i.e., the reduction in the rate of postoperative retinal detachment) has been demonstrated for vitrectomy with and without silicone oil tamponade [4] and giant retinal tear surgery [5, 6]. The work presented shows that 360° LRP can be performed using the Navilas laser system in cases where it is indicated (Table 1). Three hundred sixty-degree LRP is mostly required in conjunction with vitreoretinal procedures; in these cases, LRP is performed intraoperatively. Although using LRP in an intraoperative fashion substantially improves patient procedural tolerance, it also extends the operating time (including anesthetic time) required and not always intraoperative LRP can be done over 360°.

Sometimes postoperative, 360° LRP is not well enough tolerated by patients, since retinal photocoagulation itself is painful, and is done in the early postoperative period, thus potentially contributing to increased pain sensation (e.g., following lens contact with conjunctival sutures). However, postoperative LRP can be performed over 360° (in one or more sessions) as the subretinal fluid resolves, with no anesthetic except some topical. The use of a single-spot laser for 360° LRP is more time-consuming than other approaches, which affects tolerability of the procedure; unfortunately, there are no available data on the use of the navigated pattern laser technology for this purpose.

In the study presented, we found that the use of 360°-pattern LRP has the advantages of (1) reduced time required for achievement of the surgical goal due to reduced number and duration of LRP sessions and (2) less pain due to numerous short duration laser burns and reduced need for lens manipulation on the eye because of a wide field of view. Moreover, no additional technical difficulties were found, and the navigated pattern laser technology allows performing LRP in the amount as great as required and at any reasonable time after various retinal detachment surgeries.

We found this approach to postoperative 360°-LRP at least as effective as the two other approaches, with similar rate of redetachment in the early period after silicone oil removal. Moreover, the mean number of laser spots in postoperative 360°-pattern LRP was higher than those relevant to other methods, which can be explained by improved tolerability and treatment speed. The clinical significance of these differences in regard to prevention of retinal redetachment deserves further study.

The so-called Rapid PRP is a special feature of the navigated pattern laser (Navilas), and, with the laser pulse duration and separation as short as of 30 ms and 10 ms, respectively, the time required for the application of a 25-spot pattern is no more than 1 s, allowing to cut the laser treatment time (in panretinal laser photocoagulation) a half [10, 11]. This was further confirmed by the findings of this study, since the 360°-LRP using the slit-lamp or indirect ophthalmoscope required twice as much time (in spite of a lower total number of applied laser burns) compared to the navigated pattern laser approach. In the SL-LRP, a narrow wide field of view necessitates frequent slit-lamp and lens movements for coagulation of the next retinal location, which, along with a rather slow application of laser burns, results in a low number of placed spots and necessity for additional LRP sessions. We found patients of the IO-LRP group to have comparatively high pain scores; it was probably caused by large laser spots on the retina, since a 20-D lens was used. However, the use of a smaller laser spot in the IO-LRP group would increase the procedural time, which is already larger than that in the pattern LRP. The advantages of IO-LRP are the possibility of performing scleral depression (however, this is not necessary in the presence of CESB) and the possibility of performing the procedure in patients incapable of taking a sitting position.

A limitation of this study is the absence of data for comparison of postoperative 360° pattern LRP and intraoperative 360° LRP, with the latter being a widely used typical procedure [2–6, 8]. However, the retinal detachment rate after silicone oil removal in the pattern LRP group was similar to those reported in patients who had received intraoperative 360-degree laser retinopexy [4, 9].

Potentially, the navigated pattern approach may be used not only for 360° LRP, but also for other versions of postoperative LRP, including the LRP at the retinal tear site in meridional or circular extrascleral buckling and the LRP under conditions of pneumatic retinopexy or short-term perfluorocarbon fluid tamponade.

In conclusion, the navigated pattern approach (Navilas) to 360° LRP (a) allows improving the treatment time and pain in postoperative 360° LRP and presents no technical difficulties additional to the conventional (slit-lamp or indirect ophthalmoscope) approaches with a single-spot laser and (b) is at least as effective in achieving the surgical goal as these approaches.

## Figures and Tables

**Table 1:** Indications for postoperative laser retinopexy (and proportion of patients included in the study).

	Indications for postoperative laser retinopexy	Number of cases		
		Pattern LRP	SL-LRP	IO-LRP
(1)	Vitrectomy for rhegmatogenous retinal detachment (RRD) [4, 7, 8]	10	5	2
(2)	Silicone oil tamponade for RRD [9]	12	11	9
(3)	Circular scleral buckling for RRD [5]	14	12	11

LRP: laser retinopexy; SL-LRP: laser retinopexy using single-spot slit-lamp laser delivery; IO-LRP: laser retinopexy using single-spot indirect ophthalmoscope laser delivery.

**Table 2:** Characteristics of the study population.

	Pattern LRP	SL-LRP	IO-LRP
Patients, total	36	28	22
Age, years	50.8 ± 8.8	61.9 ± 12.4	55.1 ± 11.0
Sex, male/female	22/14	13/15	9/12
Patients with IOL	22	17	16

LRP: laser retinopexy; SL-LRP: laser retinopexy using single-spot slit-lamp laser delivery; IO-LRP: laser retinopexy using single-spot indirect ophthalmoscope laser delivery.

**Table 3:** Comparison of primary endpoints between pattern LRP, SL-LRP, and IO-LRP groups.

	Pattern LRP	SL-LRP	IO-LRP
Procedural time, minutes	12.4 ± 5.4	21.7 ± 7.6	17.0 ± 10.1
Procedural pain score	1.1 ± 0.5	1.8 ± 0.5	1.9 ± 0.5
Total number of laser burns applied	1108.7 ± 345.5	714.5 ± 219.8	408.1 ± 95.5
Number of LRP sessions	1.2 ± 0.4	2.0 ± 0.6	1.9 ± 0.7
Days after circular scleral buckling or vitrectomy	2.0 ± 1.4	3.9 ± 3.1	2.4 ± 1.9
Days after initiation of tamponade	119.8 ± 67.0	103.1 ± 54.3	88.5 ± 61.4

LRP: laser retinopexy; SL-LRP: laser retinopexy using single-spot slit-lamp laser delivery; IO-LRP: laser retinopexy using single-spot indirect ophthalmoscope laser delivery.
